# *Mycobacterium* PPE31 Contributes to Host Cell Death

**DOI:** 10.3389/fcimb.2021.629836

**Published:** 2021-04-13

**Authors:** Siyuan Feng, Zhongsi Hong, Guoliang Zhang, Jiachen Li, Guo-Bao Tian, Haibo Zhou, Xi Huang

**Affiliations:** ^1^ Center for Infection and Immunity, The Fifth Affiliated Hospital of Sun Yat-sen University, Zhongshan School of Medicine, Sun Yat-sen University, Zhuhai, China; ^2^ Key Laboratory of Tropical Disease Control, Ministry of Education, Sun Yat-sen University, Guangzhou, China; ^3^ Shenzhen Key Laboratory of Pathogen and Immunity, National Clinical Research Center for Infectious Disease, State Key Discipline of Infectious Disease, Shenzhen Third People’s Hospital, Second Hospital Affiliated to Southern University of Science and Technology, Shenzhen, China; ^4^ The Sixth Affiliated Hospital of Guangzhou Medical University, Qingyuan People’s Hospital, Qingyuan, China; ^5^ Sino-French Hoffmann Institute of Immunology, College of Basic Medical Science, Guangzhou Medical University, Guangzhou, China

**Keywords:** virulence factors, PPE31, cell death, Mycobacterium tuberculosis, JNK signaling

## Abstract

Genome scale mutagenesis identifies many genes required for mycobacterial infectivity and survival, but their contributions and mechanisms of action within the host are poorly understood. Using CRISPR interference, we created a knockdown of *ppe31^Mm^* gene in *Mycobacterium marinum* (*M. marinum*), which reduced the resistance to acid medium. To further explore the function of PPE31, the *ppe31* mutant strain was generated in *M. marinum* and *Mycobacterium tuberculosis* (*M. tuberculosis*), respectively. Macrophages infected with the *ppe31^Mm^* mutant strain caused a reduced inflammatory mediator expressions. In addition, macrophages infected with *M. marinum* Δ*ppe31^Mm^* had decreased host cell death dependent on JNK signaling. Consistent with these results, deletion of *ppe31^Mtb^* from *M. tuberculosis* increased the sensitivity to acid medium and reduced cell death in macrophages. Furthermore, we demonstrate that both *ppe31* mutants from *M. marinum* and *M. tuberculosis* resulted in reduced survival in macrophages, and the survivability of *M. marinum* was deceased in zebrafish due to loss of *ppe31^Mm^*. Our findings confirm that PPE31 as a virulence associated factor that modulates innate immune responses to mycobacterial infection.

## Introduction

Several mycobacterial species are successful intracellular pathogens of humans and other animals, and can survive and replicate within host macrophages (Houben et al., 2006; Pieters, 2008). *Mycobacterium tuberculosis*, the leading infectious killer of humans worldwide, specifically has developed a plethora of strategies to escape killing by host defense mechanisms. Under acidic conditions, *M. tuberculosis* Type VII secretion systems can cause phagolysosomal rupture (Conrad et al., 2017) and damage to the phagosomal membrane facilitates bacterial translocation into the cytosol, a process that ultimately leads to macrophage necrosis (Abdallah et al., 2011; Houben et al., 2012). In addition, necrotic cells provide an adaptive environment for proliferation/survival (Behar et al., 2010). Virulence factor used by *M. tuberculosis* manipulated macrophage death pathways, which is one of strategies to evade host immune defenses. Although the evasion of intracellular host defense by *M. tuberculosis* is crucial for host cell necrosis and bacterial dissemination, the molecular mechanisms involved remains incompletely understood.

Members of the PE/PPE protein family, which are present primarily in slowly growing mycobacteria, have been associated with virulence (Tobin and Ramakrishnan, 2008). Most PE/PPE proteins are located in the cell wall and some have been shown to modulate host cell physiology by different mechanisms (Deng et al., 2015; Yang et al., 2017). For example, *M. tuberculosis* PPE38 dampens CD8^+^ T cell responses by inhibiting macrophage MHC Class I expression (Meng et al., 2017), suggesting a unique role of PE/PPE protein to facilitate mycobacteria to escape host immunity. PPE31 belongs to the PPE subfamily and is highly expressed in *M. tuberculosis* during acidic stress, Mg^2+^ starvation and antibiotic treatment, as well as in *M. tuberculosis* infected macrophages (Walters et al., 2006; Rohde et al., 2007; Liu et al., 2016). Furthermore, an *M. tuberculosis ppe31* transposon mutant was attenuated virulence in a mouse infection model (Sassetti and Rubin, 2003), indicating a role for PPE31 in virulence; however, its precise function in pathogenesis is unknown.

In the current study, we showed that *ppe31^Mm^ (MMAR_2683)*, the *M. marinum* orthologue of *ppe31^Mtb^ (rv1807)*, was associated with mycobacterial survival in acid medium. The mutants for *ppe31^Mm^* reduced host cell death through JNK-dependent regulation of reactive oxygen species (ROS) signaling. we also confirm that deletion of *ppe31^Mtb^* in *M. tuberculosis* H37Rv decreased inflammatory cytokine expressions and reduced cell death. Consistent with these findings, PPE31 not only provides a survival advantage to *M. marinum* in macrophage, but also, was necessary for intracellular growth of *M. tuberculosis*. These findings identify PPE31 contributing to host cell death, and provide new insights into a mechanism by which PPE proteins alter host signaling to affect *M. tuberculosis* pathogenesis.

## Materials and Methods

### Ethics Statement

With the approval from Ethics Committee of Zhongshan School of Medicine on Laboratory Animal Care (reference number: 2016-159), Sun Yat-sen University, all the animal experiments were conducted based on the standard of the National Institutes of Health Guide for the Care and Use of Laboratory Animals.

### Zebrafish Infection Experiments

In order to perform microinjections, borosilicate needles were prepared. The needles were then connected to a Warner PLI-100A pump and handled using a Narishige MN-152 micromanipulator. All the injections were performed on zebrafish larvae previously anaesthetized with tricaine (Finquel; 0.02% in embryo water) and placed on a Petri dish containing a hardened solution of 1% agarose in egg water. *M. marinum* M or its isogenic strain containing pSMT3-mCherry was grown. The injection of *M. marinum* was performed at 36 h postfertilization with an injection of 300 colony-forming units (CFU) per embryo in the neural tube. After injection, embryos were transferred into fresh egg water and incubated at 28°C for 4 days before collection. Proper infection was controlled by fluorescent imaging before embryo dissociation.

### Bacterial Strains

*M. marinum* M wild-type strain and the modified vector, pPR27-GFP (ts oriM; sacB counter selection; GFP; Gent^R^) was a gift from Prof. Qian Gao, Fudan University. *M. marinum* Δ*ppe31^Mm^* and *M. marinum comp-*Δ*ppe31^Mm^* strains were generated in our laboratory. Δ*ppe31^Mm^* was generated by means of a two-step gene replacement strategy using pPR27-GFP as described previously (Pelicic et al., 1997) (**Figure S1**). Primer sequences used are listed in **Table S1**.

For construction of the complementation strains, *M. tuberculosis* homolog *ppe31 ^Mtb^* (*rv1807*) fused with a HA tag was first cloned into a pMV306-*hsp* (mycobacterial integration vector; integrates into the attB site; Kan^R^; *hsp60* promoter), and the recombinant plasmid pMV306-*hsp*-*ppe31^Mtb^*-HA was integrated into the chromosomes of the deletion strains.

To generate the *ppe31^Mtb^* knock-out in *M. tuberculosis* H37Rv strain, Δ*ppe31^Mtb^* was generated by phage specialized transduction as described previously (Jain et al., 2014). The deletion was confirmed by PCR analysis and sequencing (**Figure S4**).

### Construction of CRISPR Interference Targeting Constructs in *M. marinum*

The CRISPR interference system in *M. marinum* was constructed as previously described (Singh AK et al., 2016). Briefly, oligos targeted 3 sites of ppe31 gene were designed for the construction of sgRNAs. The oligos were annealed for the generation of double-stranded DNA inserted fragment, and the terminals were phosphorylated by T4 polynucleotide kinase. The inserted fragments were then ligated into BbsI digested pRH2521 to obtain pRH2521-sgRNA for the encoding of sgRNAs. pRH2521 harboring sgRNA targeting at gene of interest was electroporated into *M. marinum* with the expression of dCas9 encoded by pRH2502. Transformants were screened by hygromycin and kanamycin resistance agar plate.

### Analysis of *In Vitro* Response to Stress

For *M. marinum* knockdown strains, to induce the expression of the sgRNA and dCas9, different strains were grown to an OD=0.2, then the anhydrotetracycline (aTc) was added to the final concentration 200 ng/ml every 48h into 7H9-OADC medium containing hygromycin and kanamycin. Once the growth of each strain reached the OD600 = 0.5, the culture was centrifuged and the cell pellet was resuspended in a 7H9 medium (pH= 4.5) for 9 h in the presence or absence of aTc. After the treatment, ten-fold dilutions were spotted onto Middlebrook 7H10 agar containing hygromycin and kanamycin and bacteria numbers were counted after 7-9 days culture.

For *M. marinum* knockout strains, WT, Δ*ppe31^Mm^* or *comp-*Δ*ppe31^Mm^* strains were grown into optimal concentration (OD_600 _= 0.4) in 7H9 medium containing indicated antibiotics. Bacteria were then pelleted, washed three times with 7H9/Tween-80 and cells were resuspended in Middlebrook 7H9 of pH=4.5 for 9h. After treatment, the corresponding strains ten-fold dilutions were spotted onto Middlebrook 7H10 agar, and number bacteria were counted after 7-10 days culture.

For *M. tuberculosis* knockout strains, Δ*ppe31^Mtb^* or H37Rv strains were grown into optimal concentration (OD_600 _= 0.4) in 7H9 medium containing indicated antibiotic. Bacteria were then pelleted, washed three times with 7H9/Tween-80 and cells were resuspended in Middlebrook 7H9 of pH= 4.5 or pH= 5.5 for 7 days. After treatment, the corresponding strains ten-fold dilutions were spotted onto Middlebrook 7H10 agar, and bacterial enumeration was performed after 21 days post culture.

### Cell Culture

Bone marrow was isolated from the femurs and tibiae of 8 to 12-week old mice were used for the preparation of bone marrow derived macrophage (BMDMs). To induce differentiation, the cells were cultivated in DMEM containing 10% FBS, 2 mM L-glutamine, 1 mM sodium pyruvate, 100 U/ml penicillin, 100 µg/ml streptomycin, and 30% L929 conditioned medium. In DMEM supplemented with 10% fetal bovine serum (FBS) and 100 U/ml penicillin, 100 µg/ml streptomycin (GIBCO, Invitrogen). Bone marrow isolated from the femurs and tibiae of 8 to 12-week old mice were used for the preparation of BMDMs. To induce differentiation, the cells were cultivated in DMEM containing 10% FBS, 2 mM L-glutamine, 1 mM sodium pyruvate, 100 U/ml penicillin, 100 µg/ml streptomycin, and 30% L929 conditioned medium. Non-adherent cells were washed by PBS after 24 h and cultured for 7 days.

### Macrophage Infection Study

BMDMs were grown in DMEM containing 10% FBS and 10% L929 conditioned medium. Cells were allowed to adhere in a 24-well plate for 24 h at 37°C at a density of 3×10^5^ cells/well in 1 ml at 37°C under an atmosphere containing 5% CO_2_. For infections of Raw264.7 cells and BMDMs, mycobacteria were cultivated at 30°C (for *M. marinum*) or 37°C (for *M. tuberculosis*) in 7H9 supplemented with 10%OADC and 0.05% Tween 80 to reach mid-log phase growth. Bacterial culture pellets were re-suspended with 1 ml of PBS. Mycobacterium pellets were homogenized to generate single cell suspension, and the aggregates were removed by a short spin for 1 min at 1200 rpm. The mycobacterium suspensions were diluted by DMEM. BMDMs were then infected at the indicated multiplicity of infection (MOI). BMDMs were incubated for 1h at 30°C with 5% CO_2_ to induce phagocytosis. Extracellular bacteria were washed out with PBS for three times. To quantify the number of internalized bacteria, BMDMs were incubated for an additional 48 hours under the same condition. At indicated time point, BMDMs were lysed by 0.1% Triton X-100 in PBS and serial dilutions of the lysates were plated on 7H10-OADC. Colony forming units (CFU) were counted after 8–10 days of incubation at 30°C for *M. marinum* and after 21 days of incubation at 37°C for *M. tuberculosis*, respectively.

### Real-Time PCR and PCR Analysis

Total RNA was extracted as previously described (Wu et al., 2013). Quantitative real-time PCR was performed in Bio-Rad CFX96 real-time detection system. For mammalian cells, relative mRNA expression levels were calculated by normalization to *β-actin*. For mycobacterial cells, relative mRNA expression levels were calculated by normalization to *sigA*.

### Cytokine Measurements

Cytokine expression was measured in culture supernatants harvested from *M. tuberculosis*-infected Raw264.7 macrophages at 24 h after infection. TNF and IL-6 levels were measured by ELISA kits (R&D Systems) according to the manufacturer’s instructions.

### Western Blot Analysis

Cells were washed three times with ice-cold PBS and lysed by lysis buffer containing 1 mM phenylmethylsulfonyl fluoride, 1% (vol/vol) protease inhibitor cocktail (Sigma) and 1 mM DTT, followed by centrifugation at 12,000g for 5 minutes. Equal amounts (20 µg) of cell lysates were loaded for SDS-PAGE and transferred to PVDF membranes. Membranes were blocked by 5% BSA in PBST and incubated overnight with the respective primary antibodies at 4°C. Antibodies against phosphorylated MAPKs (CST) were diluted in 1: 1000 ratio. The membranes were incubated at room temperature for 1 h with appropriate HRP-conjugated secondary antibodies (1: 3000 dilution). The immunoblots were further visualized by reacting with Plus-ECL (PerkinElmer, Shelton, CA) according to the manufacturer’s protocol.

### Immunofluorescence Microscopy

Immunostaining was conducted as previously described (Wang et al., 2013). Briefly, cells were seeded on coverslips, and treated, then fixed, thereafter permeabilized and blocked. Cells were then incubated with primary antibodies at 4°C overnight, and then with secondary antibodies for 1 h at room temperature. Additionally, nuclei were labeled by 4,6-diamidino-2-phenylindole (DAPI). Coverslips were mounted with ProLong Gold anti-fade reagent (Invitrogen), and images were captured by Olympus BX53 fluorescence microscope (Olympus Corporation, Tokyo, Japan).

### Cell Death Assay

Macrophages were stained with propidium iodide (PI) (KeyGEN BioTECH) for 10 minutes and sorted by flow cytometry (at least 8,000 cells acquired, BD Accuri C6). For TUNEL staining, cells were fixed by 4% paraformaldehyde overnight prior to staining. Detection of cell death was conducted under manufacturer’s instructions (Roche) and examined by fluorescence microscopy.

### Cytotoxicity Assays

Cytosolic lactate dehydrogenase (LDH) release (OD490nm) was measured using CytoTox 96 assay (Promega) to monitor cytotoxicity. Lysing of infected cells with 1% Triton-X enabled maximum LDH release, while supernatants of control cells lysates were assessed for spontaneous LDH release. Measurement of cytotoxicity was carried out by calculating the percentage of LDH release net change as shown in the following formula: (test LDH release - spontaneous release)/(maximal release - spontaneous release) ×100.

### Measurement of ROS Generation

The measurement of ROS levels in BMDMs were conducted as previously described (Wang et al., 2014). At the indicated time points after infection, cells were harvested and stained with 10μM CM-H2DCFDA (Invitrogen) for 30 minutes at 37°C in basic DMEM and then washed twice with PBS. Following workflow were analyzed by flow cytometry (at least 8,000 cells acquired, BD Accuri C6).

### Statistical Analysis

Statistical analysis was performed using Prism (version 6.0c; GraphPad Software). Statistical significance of paired comparison data was assessed by paired student’s *t*-test. Statistical significance of data with multiple confounding factors were assessed by analysis of variance (ANOVA). A *p* value of 0.05 or lower was considered of statistical significance.

## Results

### *ppe31^Mm^* Is Required for *M. marinum* Resistance to Acid Medium

**Figure 1:**
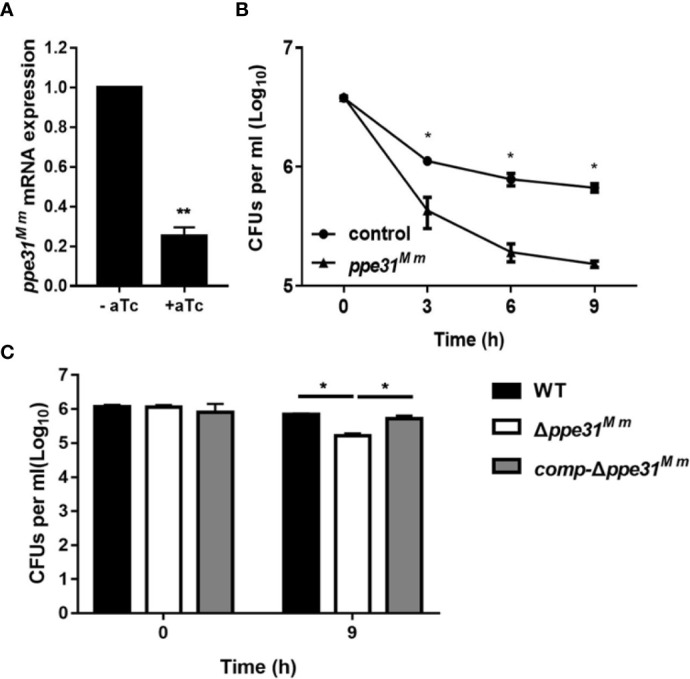
PPE31^Mm^ was required for the resistance of *M. marinum* to acid medium *in vitro*. **(A)** CRISPR interference has been performed for knockdown of *ppe31^Mm^*. Expression of *ppe31^Mm^* determined by RT-qPCR with or without induction of aTc. **(B)** *M. marinum* contain dCas9 and sgRNA targeting *ppe31^Mm^* were exposed to 7H9 medium of pH4.5 with or without (control) induction of aTc. The bacterial survival was monitored by CFU counting at the indicated time. **(C)** WT, Δ*ppe31^Mm^*, or *comp-*Δ*ppe31^Mm^* were exposed to 7H9 medium of pH4.5 at 30°C. The bacterial survival was monitored by CFU counting for 9h. Data are shown as mean ± S.E.M. of three independent experiments. *p < 0.05, **p < 0.01.

According to previous studies, the *ppe31* is upregulated in *M. tuberculosis* during acidic stress (Walters et al., 2006; Rohde et al., 2007; Goodsmith et al., 2015). We sought to probe the phenotype of PPE31 through knock down *ppe31^Mm^* gene in *M. marinum* by means of CRISPR interference (CRISPRi) (Singh AK et al., 2016). To do this, silencing efficiency was measured by real-time PCR and showed ~80% decreased expression of *ppe31^Mm^* (**Figure 1A**). We found that the decreased *ppe31^Mm^* expression significantly reduced the survival of *M. marinum* in 7H9 medium of low pH compared to the strain without induction of aTc (control) (**Figure 1B**). To further explore the function of *ppe31^Mm^*, we constructed a *ppe31^Mm^* deletion mutant (Δ*ppe31^Mm^*) in *M. marinum* by substituting the *ppe31^Mm^* gene with a cassette coding for hygromycin resistance (**Figure S1**) and used this strain to investigate the impact of the PPE31 on its survivability in acid medium, and we also constructed a complementation strain *comp-*Δ*ppe31^Mm^*, which integrated *M. tuberculosis ppe31^Mtb^* fused with a HA tag sequence into the chromosomes of *M. marinum* Δ*ppe31^Mm^* strain. WT, Δ*ppe31^Mm^* or *comp-*Δ*ppe31^Mm^* were cultured in 7H9 of pH4.5, the result showed that when compared to WT or *comp-*Δ*ppe31^Mm^*, *M. marinum* lack of *ppe31^Mm^* significantly reduced the resistance to acid medium (**Figure 1C**).

### *M. marinum* Δ*ppe31^Mm^* Infection Down-Regulates Proinflammatory Cytokine Expression and ROS Generation in BMDMs

**Figure 2:**
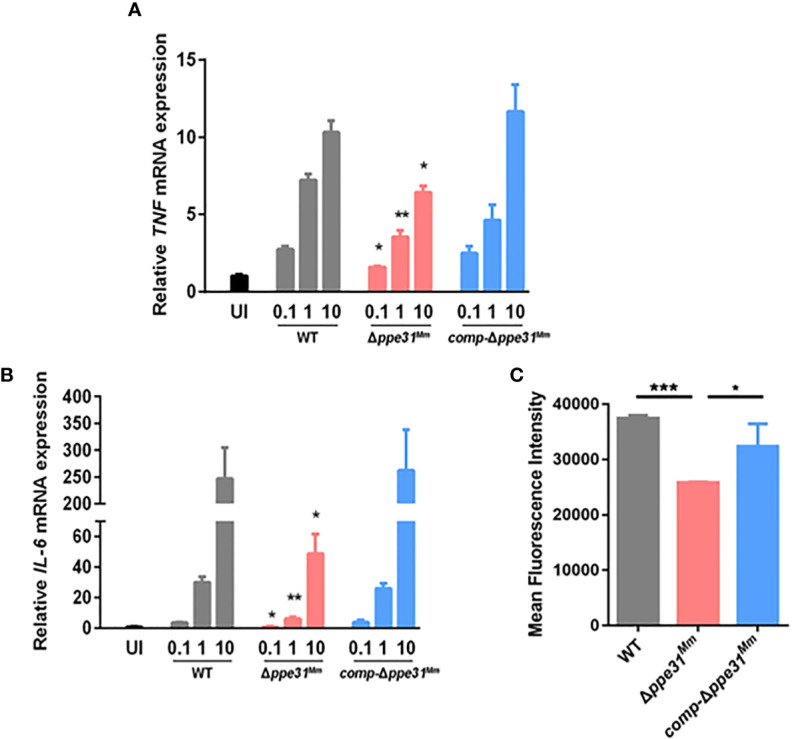
Δ*ppe31^Mm^* infection fails to partly induce the expression of proinflammatory cytokines and ROS generation in BMDMs. BMDMs were infected with WT, Δ*ppe31^Mm^*, or *comp-*Δ*ppe31^Mm^* at different MOIs (0.1, 1 and 10) for 6 h. The level expressions of inflammatory cytokines, TNF **(A)** and IL-6 **(B)** were assessed by RT-qPCR. **(C)** BMDMs were stimulated with WT, Δ*ppe31^Mm^ or comp-*Δ*ppe31^Mm^* for 30 min. Cells were then labeled with DCFH-DA that were used detecting cytosolic ROS, and were analyzed for cytosolic ROS levels using flow cytometry. Data are shown as mean ± S.E.M. of three independent experiments. *p < 0.05, **p < 0.01, ***p < 0.001. WT, wild-type *M. marinum*; UI, uninfected.

PE/PPE family proteins, which are mainly located in the cell surface, interact with innate receptors in the phagocytes to modulate inflammatory mediators such as IL-6, IL-12p40 and TNF, and the generation of reactive oxygen species (ROS) (Cadieux et al., 2011; Deng et al., 2015; Deng et al., 2016). The expression of proinflammatory cytokines were examined and TNF and IL-6 mRNA expression was decreased in the BMDMs infected with Δ*ppe31^Mm^* strain compared to WT or *comp-*Δ*ppe31^Mm^* although all strains showed increased cytokine production with increased bacterial inocula (**Figures 2A, B**). These findings suggest a regulatory role for *ppe31^Mm^* in proinflammatory cytokine expression in macrophages infected with *M. marinum*.

Since proinflammatory cytokine production was strongly related with ROS generation, our hypothesis is that ROS levels differed between cells infected with WT, *comp-*Δ*ppe31^Mm^* and Δ*ppe31^Mm^*, strains of *M. marinum.* The production of ROS was measured by flow cytometry, using 2,7’-dichlorofluorescein-diacetate (DCFH-DA). Compared with BMDMs infected with WT or *comp-*Δ*ppe31^Mm^* strains, cells infected with Δ*ppe31^Mm^* displayed significantly decreased of intracellular DCFH-DA fluorescence (**Figure 2A**). Additionally, we explored whether there is a relationship between ROS generation and the inflammatory response. When we inhibited the generation of ROS by using DPI, a reactive oxygen species inhibitor, we observed that the expression of inflammatory cytokines was significantly reduced (**Figure S2**). Altogether, these data suggest that PPE31^Mm^ is involved in the modulation of inflammatory mediators in BMDMs.

### Involvement of PPE31^Mm^ in Caspase-Independent Cell Death

**Figure 3:**
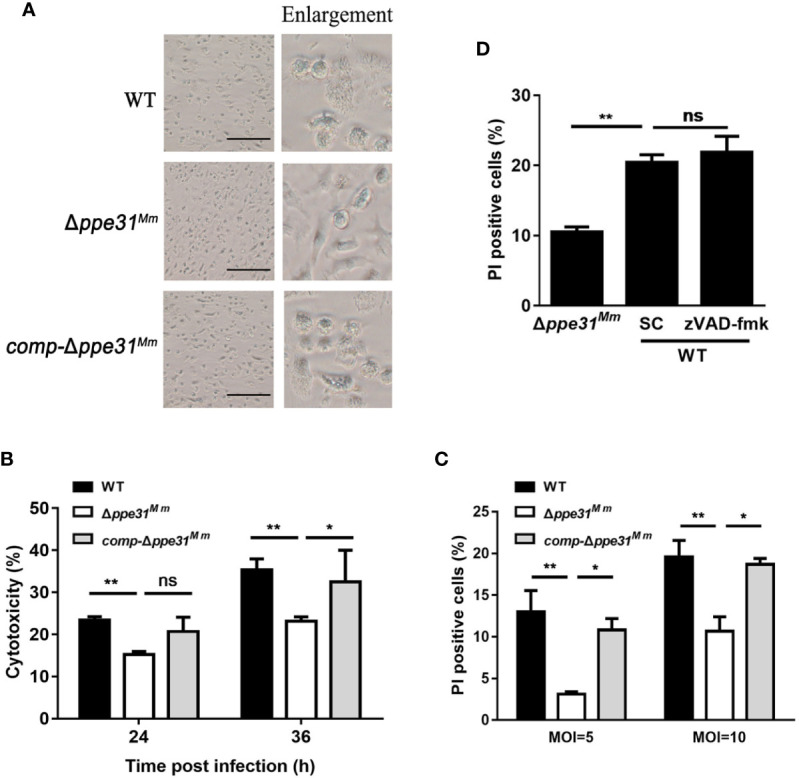
Macrophages infected with Δ*ppe31^Mm^* show reduced caspase- independent cell death. **(A)** BMDMs were infected with WT, Δ*ppe31^Mm^*, or *comp-*Δ*ppe31^Mm^* (MOI = 10) for 24h for analysis of morphological changes. Scale bar in 100 μm. **(B)** BMDMs were infected with WT, Δ*ppe31^Mm^*, or *comp-*Δ*ppe31^Mm^* (MOI = 10). The cytotoxicity was assessed by LDH release assay at indicated times. **(C)** BMDMs were infected with either WT, Δ*ppe31^Mm^*, or *comp-*Δ*ppe31^Mm^* (MOI = 10 or 5) for 24 h, and cell death was detected by PI staining and then examined by flow cytometry. **(D)** BMDMs were infected with WT, Δ*ppe31^Mm^* or *comp-*Δ*ppe31^Mm^* (MOI = 10) in the presence or absence of Z-VAD-FMK (20 μM), a caspase inhibitor, then cells were stained with PI and then examined by flow cytometry. Data are shown as mean ± S.E.M. of three independent experiments. *p < 0.05, **p < 0.01. WT, wild-type *M. marinum*; UI, uninfected; SC, solvent control (0.1% DMSO); ns, no significant.

During infection, we observed that at 24h post-infection, morphological changes associated with cell death in WT or *comp-*Δ*ppe31^Mm^-*infected cells, but not in Δ*ppe31^Mm^* (**Figure 3A**), indicating that wild-type *M. marinum* disrupted the integrity of cell membrane, a process that is critical for necrosis. We next examined the cytotoxicity of Δ*ppe31^Mm^*, after infection with these three strains at an MOI of 10, a significant decrease in cell cytotoxicity at 24h and 36h post-infection was observed in Δ*ppe31^Mm^*-infected BMDMs, whereas WT and *comp-*Δ*ppe31^Mm^*-infected cells displayed higher cytotoxicity (**Figure 3B**). To determine whether PPE31 can regulate host cell death in macrophages, we further assessed whether necrosis played a role in cell death induced by *M. marinum*. Using propidium iodide (PI) staining, we found that *ppe31^Mm^* deficiency causes less cell death of infected BMDM cells at two different MOI (**Figure 3C**). To further examine the mechanism by which Δ*ppe31^Mm^*-infected cells induce cell death, we cultured BMDM cells infected with WT or Δ*ppe31^Mm^* (MOI = 10) in the presence or absence of the caspase inhibitor Z-VAD-FMK. We found that this inhibitor could not block WT-mediated cell death (**Figure 3D**). Collectively, these results suggest that Δ*ppe31^Mm^* is associated with decreased cell cytotoxicity and reduced caspase-independent cell death.

### Infection With Δ*ppe31^Mm^* Reduces Cell Death Through JNK Signaling

**Figure 4:**
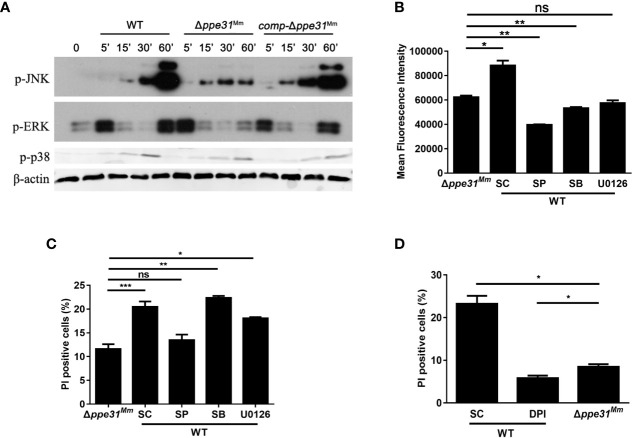
Infection with Δ*ppe31^Mm^* reduces cell death through JNK-dependent signaling. **(A)** BMDMs were infected with WT, Δ*ppe31^Mm^* or *comp-*Δ*ppe31^Mm^* (MOI = 10) for the indicated periods of time, and then subjected to Western blot analysis using antibodies raised to p-ERK1/2, p-p38, p-JNK, and β-actin. **(B)** BMDMs were pretreated with the following MAPK signaling pathways inhibitors U0126 (20 μM), SB203580 (SB; 10 μM), or SP600125 (SP; 20 μM) for 1h, and then infected with WT, Δ*ppe31^Mm^* or *comp-*Δ*ppe31^Mm^* for 30 min, Cells were then incubated with DCFH-DA and analyzed immediately for ROS generation by flow cytometry. **(C)** BMDMs were pretreated with U0126 (20 μM), SB (10 μM), or SP (20 μM) for 1h, and then infected with WT, Δ*ppe31^Mm^* or *comp-*Δ*ppe31^Mm^* for 24h, cells were stained with PI and then examined by flow cytometry. **(D)** BMDMs were infected with WT, Δ*ppe31^Mm^* or *comp-*Δ*ppe31^Mm^* (MOI = 10) in the presence or absence of DPI (10 μM). After 24h, cells were then stained with PI and analyzed immediately for cell death using flow cytometry. Data are shown as mean ± S.E.M. of three independent experiments. *p < 0.05, **p < 0.01, ***p < 0.001. WT, wild-type; *M, marinum*; UI, uninfected; SC, solvent control (0.1% DMSO); ns, no significant.

MAPK signaling pathways are critical in oxidative stress-mediated cell death during mycobacterial infection (Kim et al., 2012). To investigate whether MAPK signaling pathways are involved in PPE31^Mm^-mediated cell death, BMDM were infected with WT, *comp-*Δ*ppe31^Mm^* or Δ*ppe31^Mm^* strains. We found the activation kinetics of phosphorylated p38 and ERK1/2 between cells infected with these three strains was no significant difference. On the contrary, a decrease in JNK/SAPK phosphorylation was observed in cells infected with *M. marinum* Δ*ppe31^Mm^* when compared with cells infected with WT or *comp-*Δ*ppe31^Mm^* (**Figure 4A**). These results indicate that JNK signaling may be involved in PPE31^Mm^ mediated cell death.

To investigate the relationship between MAPK signaling pathway and PPE31^Mm^-mediated ROS generation and cell death, BMDMs were pretreated with specific inhibitors of JNK, p38, and MEK for 1 h prior to infection with WT or Δ*ppe31^Mm^*. All three inhibitors significantly decreased ROS generation, indicating that all three kinases in MAPK signaling pathways are involved in the regulation of ROS generation in response to mycobacterial infection (**Figure 4B**). In addition, inhibition of JNK and ERK signaling pathway, but not p38, reduced macrophage death infected with WT, and JNK signaling was dominant in PPE31^Mm^-mediated cell death (**Figure 4C**). We also examined the link between MAPK signaling pathways and the mRNA of inflammatory cytokines. Inhibition of all three kinases in MAPK signaling pathway, reduced PPE31^Mm^-induced mRNA of TNF and IL-6 in a dose-dependent manner (**Figure S3**).

Furthermore, we examined the relationship between ROS generation and macrophage survival. BMDMs were infected WT, *comp-*Δ*ppe31^Mm^* or Δ*ppe31^Mm^*, for 24 h in the presence or absence of DPI. Inhibition of ROS generation significantly decreased PPE31^Mm^-induced cell death (**Figure 4D**). Together, these data suggest that PPE31^Mm^ modulates macrophage survival and inflammatory response in *M. marinum* infected cells through JNK-dependent regulation of ROS signaling.

### Deletion of *ppe31^Mtb^* From *M. tuberculosis* H37Rv Decreases Inflammatory Mediator Expressions and Reduces Host Cell Death

**Figure 5:**
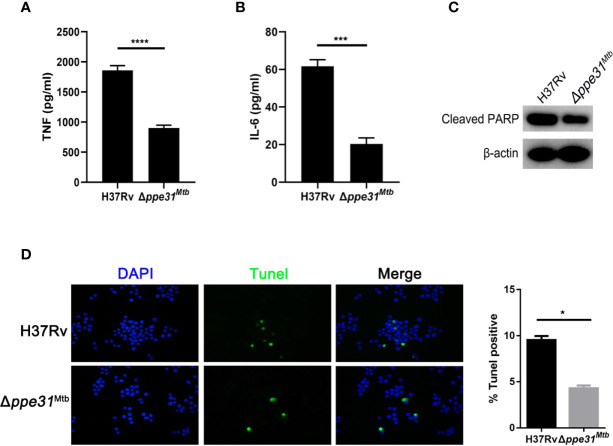
Mutants for *ppe31^Mtb^* decreases the inflammatory cytokine expressions and reduces host cell death. RAW264.7 cells were infected with H37Rv or Δ*ppe31^Mtb^* (MOIs=10) for 24 h. The supernatants were collected, sterile-filtered, and assayed for TNF **(A)** and IL-6 **(B)**. **(C)** RAW264.7 were infected with H37Rv or Δ*ppe31^Mtb^* (MOI = 10) for 24h, and then subjected to Western blot analysis using antibodies raised to cleaved PARP and β-actin. **(D)** RAW264.7 were infected with H37Rv or Δ*ppe31^Mtb^* (MOI = 10) for 24h, and cell death was detected by TUNEL staining and then examined by confocal microscope. Data are shown as mean ± S.E.M. of three independent experiments. *p<0.05, ***p<0.001, ****p<0.00001. UI, uninfected; M, marker.

Given the observation that *M. marinum* PPE31 contributed to control host immune response, we speculated that *M. tuberculosis* PPE31 may exhibit similar phenotype and functions. To do it, Δ*ppe31^Mtb^* was generated in *M. tuberculosis* H37Rv strain by recombineering (**Figure S4**). Next, *M. tuberculosis* H37Rv or Δ*ppe31^Mtb^* was cultured in 7H9 containing different gradient pH for 7 days. When compared to H37Rv, we found that, the survival rate of Δ*ppe31^Mtb^* was significantly decreased (**Figure S5C**). Additionally, assessment of transcript levels for TNF and IL-6 in infected macrophages showed that the expression of TNF and IL-6 was significantly decreased in Δ*ppe31^Mtb^* infected macrophages when compared to wild-type strains (**Figures 5A, B** and **Figures S5A, B**). Furthermore, RAW264.7 cells were infected with H37Rv and Δ*ppe31^Mtb^* and stained for genomic DNA fragmentation using the TUNEL assay. We found that *ppe31^Mtb^* deficiency in *M. tuberculosis* reduced host cell death during infection (**Figure 5D**). Notably, a decrease in cleaved PARP was observed in macrophages infected with Δ*ppe31^Mtb^* when compared with cells infected with H37Rv (**Figure 5C**).These results indicate that PPE31^Mtb^ was also important and indispensable to regulate innate immune responses to *M. tuberculosis* infection.

### PPE31 Is Required for Mycobacteria Survival in Macrophage and in Zebrafish

**Figure 6:**
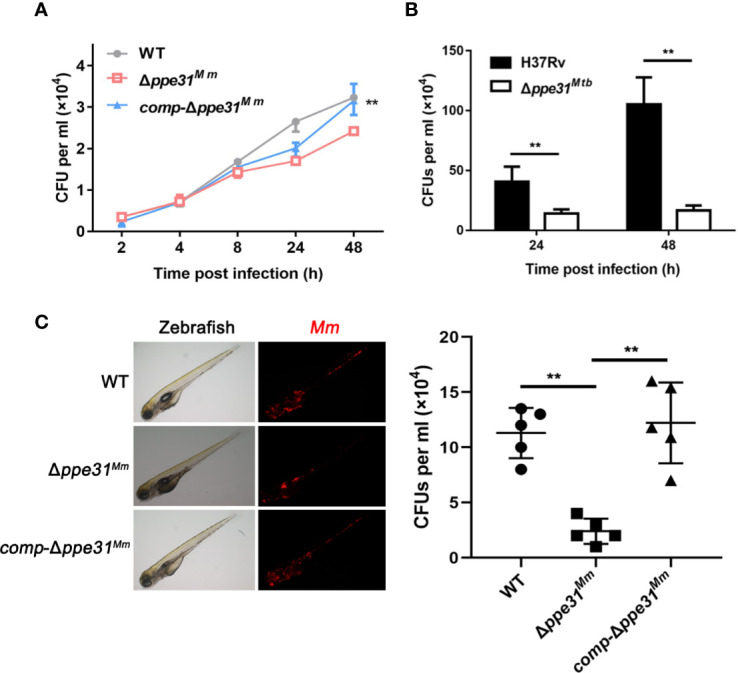
PPE31 promotes mycobacteria survival in macrophage and in zebrafish. **(A)** WT, Δ*ppe31^Mm^* or *comp-*Δ*ppe31^Mm^*-infected BMDMs were analyzed for number of surviving internalized bacilli by CFU counting at the indicated time. **(B)** H37Rv or Δ*ppe31^Mtb^*-infected RAW264.7 cells were analyzed for number of surviving internalized bacilli by CFU counting at the indicated time. **(C)** Bacterial survival advantage in the infected zebrafish. Three hundred CFU of *M. marinum* strains were injected to reach a comparable infection level at 4 dpi. Representative burden pictures and representative burden analysis derive from larvae collected at 4 dpi. Each point in represents 1 infected larva from a representative pool. Scale bar in 200 μm. Data are shown as mean ± S.E.M. of three independent experiments. **p < 0.01. WT, wild-type *M. marinum*; UI, uninfected.

Based on the above data, we hypothesize that PPE31 is essential for mycobacteria survival in macrophage. To confirm this, RAW264.7 cells were infected with WT, Δ*ppe31^Mm^* or *comp-*Δ*ppe31^Mm^* strains of *M. marinum* and the surviving intracellular bacteria were assessed at 2, 4, 8, 24, and 48h after infection. We found deletion of *ppe31^Mm^* reduced the number of surviving mycobacteria at 48h in macrophages (**Figure 6A**). In addition, survival of H37Rv and Δ*ppe31^Mtb^* were assessed by CFU in macrophages. We found that *ppe31^Mtb^* deficiency significantly decreased its survival in macrophages when compared to H37Rv (**Figure 6B**), these results were consistent with the earlier observation in mice (Sassetti and Rubin, 2003). Furthermore, zebrafish larvae were infected with the WT or Δ*ppe31^Mm^* strains. A significant reduction in the virulence of Δ*ppe31^Mm^* was observed when compared with WT (**Figure 6C**).

Overall, these results demonstrated that PPE31 enhances the intracellular survivability of mycobacteria.

## Discussion

*M. marinum* is genetically related to *M. tuberculosis* (Tobin and Ramakrishnan, 2008). Both of them share the virulence related Type VII secretion systems ESX-1 and ESX-5 (Thi et al., 2013; Singh VK et al., 2016). ESX-1 secretion systems of pathogenic mycobacteria secrete the effector proteins, including ESAT-6, which are required for the disruption of the membrane integrity of the mycobacteria containing phagosome (Conrad et al., 2017; Augenstreich et al., 2017). This was followed by the translocation of mycobacteria to the cytosol of host cells (Simeone et al., 2012). Although disruption of ESX-5 has no effect on the translocation of mycobacteria from phagolysosome to cytosol, the effector proteins secreted by ESX-5 also induce a caspase-independent cell death after translocation has taken place (Abdallah et al., 2011). Intriguingly, accumulated evidences have shown that the ESX-5 substrates, PE/PPE family proteins, are involved in the evasion of host cell immune responses (Saini et al., 2016; Camassa et al., 2017).

The important environment factor of mycobacteria containing phagosome is the pH value. During *M. tuberculosis* infection, the pH value is ranging from 6.2 within the phagosome of immature macrophage to 4.5 in the phagolysosome following INF-γ activation (Machado et al., 2019). Here, we first demonstrated that PPE31 is required for survival under acidic condition *in vitro*. It is not surprising that *ppe31^Mtb^* partly complemented the phenotype of *M. marinum* lack of *ppe31^Mm^*, due to the following factors: (i) PPE31 of *M. marinum* is 71% identical to PPE31 of *M. tuberculosis*, (ii) *M. marinum* is closely related to *M. tuberculosis* in its pathogenicity as well as genetically. A previous study reported that PPE38 shared 73% identity in amino acid sequence to MMAR_3661, complemented the phenotype of Δ*MMAR_3661* (Dong et al., 2012).

Next, we examined the role of PPE31^Mm^ in immune regulation. We showed that a *M. marinum* Δ*ppe31^Mm^* provoked reduced expression of inflammatory cytokine and ROS generation during infection of phagocytic cells. Recent reports have emphasized that several PE/PPE family proteins are potent inducers of cytokines, including TNF and IL-6. For example, PE11-transformed *M. smegmatis*-infected mice induce high levels of TNF and necrotic cell death of macrophage (Deng et al., 2015; Singh P et al., 2016). TNF constitutes a critical host defense against tuberculosis, although excessive of TNF induces necrosis *via* mitochondrial reactive oxygen species in macrophages during *M. marinum* infection (Roca and Ramakrishnan, 2013). Based on the decreased cytokine production in the Δ*ppe31^Mm^* strain, we hypothesized that PPE31 may affect the survival of *M. marinum*-infected cells. Consistent with our hypothesis, we observed prominent signs of necrosis in macrophages infected with WT strain, such as lytic plasma membranes and lactate dehydrogenase release, but these were not seen in cells infected with a Δ*ppe31^Mm^* strain. Interestingly, this type of cell death was not inhibited by the inhibitor of caspase. These results suggested that PPE31 induced caspase-independent cell death during *M. marinum* infection.

Recent studies have shown that MAPK signaling pathways, especially JNK signaling, contribute to the induction of cell death by *M. tuberculosis*. Based on this, and given the closely relationship between ROS and cell death, our data comparing wild-type to Δ*ppe31^Mm^* show that JNK signaling is involved in macrophage necrosis in cells infected with *M. marinum*. *M. tuberculosis* Eis protein suppresses host immune defenses by negatively regulating cell death through JNK-dependent inhibition of ROS generation (Kim et al., 2012). Moreover, the *Streptococcus pyogenes* Toxin triggers a form of programmed necrosis dependent on JNK signaling in *Streptococcus pyogenes* infected cells (Chandrasekaran and Caparon, 2016). Our data demonstrated that phosphorylated JNK was also involved in PPE31^Mm^-induced ROS generation and cell death. Previous work has implicated mycobacterial factors in programmed necrosis *via* mitochondrial ROS (Miller et al., 2010; Mohanty et al., 2016). Our results demonstrate that DPI, an NADPH oxidase inhibitor, significantly decreased PPE31^Mm^-induced cell death. This supports the idea that phosphorylated JNK increases oxidative stress and contributes to the induction of host cell death during *M. tuberculosis* infection (Shin et al., 2010; Chandrasekaran and Caparon, 2016).

To strengthen the proposition that PPE31 is a virulence factor that modulates innate immune responses during mycobacterial infection, *M. tuberculosis ppe31^Mtb^* mutant was constructed by specialized-phage transduction. Given the relationship between *M. marinum* and *M. tuberculosis* genetically, our data reported that *ppe31^Mtb^* is also required for *M. tuberculosis* survival in acid medium. However, we did not observe macrophages undergoing a lytic death at early stages of infection. In contrast, we observed a decreased cleaved PARP, a maker of apoptosis, in macrophage infected with Δ*pppe31^Mtb^*. Since several studies described that apoptosis is induced exclusively by virulent *M. tuberculosis* strains in marine macrophage (Derrick and Morris, 2007; Aguilo et al., 2014), indicating that the mechanism by which *M. tuberculosis* induces host cell death is different from *M. marinum* at early stage of infection. It should be noted that a delayed phagosomal rupture caused by *M. tuberculosis* infection was observed after 48 hours, but *M. marinum* escaped from phagosome within 2–4 hours (Abdallah et al., 2011).

It has been reported that PPE proteins form a heterodimer with PE proteins, and that secretion of PPE protein by ESX-5 is dependent on PE protein (Tundup et al., 2008; Tiwari et al., 2014; Tiwari et al., 2015; Chen et al., 2017), such as PE25/PPE41. Many different groups have shown that the expression of *pe20* and *ppe31* in *M. tuberculosis* are significantly increased under adverse stress (Walters et al., 2006; Rohde et al., 2007; Provvedi et al., 2009; Liu et al., 2016), indicating a close relationship between PE20 and PPE31. However, PE20 or its homologue gene is absent in *M. marinum*. Therefore, it is possible that PE20 is dispensable for mycobacterial virulence, at least in *M. marinum*. On the other hand, the substrate of ESX-5 was involved in the secretion phenotype of PE_PGRS proteins in *M. marinum*. Very recently, a study by Ates and colleagues reported that the secretion of ESX-5 substrates, including PPE-MPTR and PE_PGRS, was completely inhibited by genetic disruption of *ppe38* and this phenotype has been already confirmed both in *M. marinum* and *M. tuberculosis* (Ates et al., 2018). In the light of these results linking PPE31 to ESX-5, it is plausible that PPE31, which might be transported by ESX-5, partly increased the cell envelope integrity in *M. tuberculosis*. Further studies will be necessary to address the relationship between PPE31 and ESX-5.

Finally, we considered the role of PPE31 in intracellular survival of bacteria. Transposon mutagenesis previously showed that *M. tuberculosis ppe31::tn* caused significant attenuation in mice (Sassetti and Rubin, 2003). Our results showing shortened survival in macrophages of *M. marinum ppe31^Mm^* are consistent with this *M. tuberculosis* data *in vivo.* Moreover, *M. tuberculosis* Δ*ppe31^Mtb^* were also attenuated in macrophages. Several mechanisms could be involved in this observation; Firstly, TNF excess induced host cell necrosis is detrimental to bacterial clearance. Secondly, phagosome maturation is a vital strategy to destroy engulfed invading microorganisms. Finally, mycobacteria escape from the phagosome to access the cytosol. Then cytosolic mycobacteria are able to replicate and cause death. This suggests that an important role of PPE31 in mycobacterial survival during infection.

In summary, our results identify mycobacterial PPE31 as an important factor contributing to the modulation of host cell immune response. Our data show that this cell envelope localized protein modulates JNK signaling pathways in host cells to alter cytokine profiles and facilitate mycobacterial survival. Additional mechanisms by which PPE31 mediates the host-pathogen interaction will require further investigation.

## Data Availability Statement

The original contributions presented in the study are included in the article/**Supplementary Material**. Further inquiries can be directed to the corresponding authors.

## Ethics Statement

The animal study was reviewed and approved by Ethics Committee of Zhongshan School of Medicine on Laboratory Animal Care (reference number: 2016-159), Sun Yat-sen University.

## Author Contributions

XH and HZ conceived the study and supervised global data analysis. SF, ZH, and GZ designed and performed experiments, analyzed data, and co-wrote paper. ZH and GZ performed construction of deletion of *ppe31* gene in mycobacteria. JL performed the intracellular fitness experiment. G-BT contributed to data interpretation and wrote the manuscript. All authors contributed to the article and approved the submitted version.

## Funding

This work was supported by National Science and Technology Key Projects for Major Infectious Diseases (2017ZX10302301-002), National Natural Science Foundation of China (31470877), Development Project of Foshan Fourth People’s Hospital (FSSYKF-2020003 and FSSYKF-2020017), and Guangzhou Science and Technology Planning Project (201704020226 and 201604020006).

## Conflict of Interest

The authors declare that the research was conducted in the absence of any commercial or financial relationships that could be construed as a potential conflict of interest.

## Supplementary Material

Supplementary Figure 1 Construction of the *ppe31^Mm^* mutant. Schematic representation of the strategy used for the generation of *ppe31^Mm^* gene replacement mutant **(A)**. Confirmation of *ppe31 ^Mm^* disruption in mutant. PCR amplifications were performed using specific primers. The result shows the PCR amplification gives a product of 2.2kb for wildtype *M. marinum* and 1kb for Δ*ppe31^Mm^* **(B)**.

Supplementary Figure 2 PPE31 significantly increased the expression of inflammatory cytokines *via* reactive oxygen species in BMDMs. BMDMs were infected with WT, Δ*ppe31^Mm^* or *comp-*Δ*ppe31^Mm^* in the presence or absence in DPI (10μM). After 6h, the level of inflammatory cytokines was assessed by RT-qPCR for expressions of TNF-α and IL-6. WT, wild-type *M. marinum*, **p* < 0.05, ***p* < 0.01. Data are shown as mean ± S.E.M. of three independent experiments. **p* < 0.05, ***p* < 0.01.

Supplementary Figure 3 PPE31 significantly increased the expression of inflammatory cytokines through JNK-dependent signaling. BMDMs were pretreated with U0126 (5, 10, 20 mM), SB203580 (SB; 1, 5, 10 mM), or SP600125 (SP; 5, 10, 20 mM) for 1h, and then infected with Δ*ppe31^Mm^* for1h, washed three times by PBS. After 6h, the level of inflammatory cytokines was assessed by real time PCR for expressions of TNF-α and IL-6.

Supplementary Figure 4 Generation of *ppe31^Mtb^* gene replacement mutant. **(A)** Generation of *M. tuberculosis ppe31^Mtb^* gene replacement mutant. Schematic representation of the strategy used for the generation of *ppe31^Mtb^* gene replacement mutant. **(B)** Confirmation of *ppe31^Mtb^* disruption in mutant. PCR amplifications were performed using specific primers, using genomic DNA of H37Rv and Δ*ppe31^Mtb^* mutant. The result shows the PCR amplification with Rv ppe31-LL and Rv ppe31-RR pair, which gives a product of 3.2kb for H37Rv and 5.6kb for Δ*ppe31*.

Supplementary Figure 5 RAW264.7 cells were infected with H37Rv or Δppe31Mtb (MOIs=10) for 6 h. The level expressions of inflammatory cytokines, TNF **(A)** and IL-6 **(B)** were assessed by RT-qPCR. The sensitivity of *M. tuberculosis* strains to acid condition. The bacteria grown to mid-log phase were collected by centrifugation and resuspended to the OD600 of 0.5 in 5 ml 7H9 (pH = 4.5 or 5.5). The results are percent survival post treatment with 7H9 medium (pH =4.5 or 5.5) for 7d **(C)**. Data are shown as mean ± S.E.M. of three independent experiments. **p*<0.05. UI, uninfected; M, marker.
