# Supplementary figures and images for: Mycobacterium PPE31 Contributes to Host Cell Death
Source: Front Cell Infect Microbiol. 2021 Apr 13;11:629836. doi: 10.3389/fcimb.2021.629836 (PMC8078103; doi:10.3389/fcimb.2021.629836)

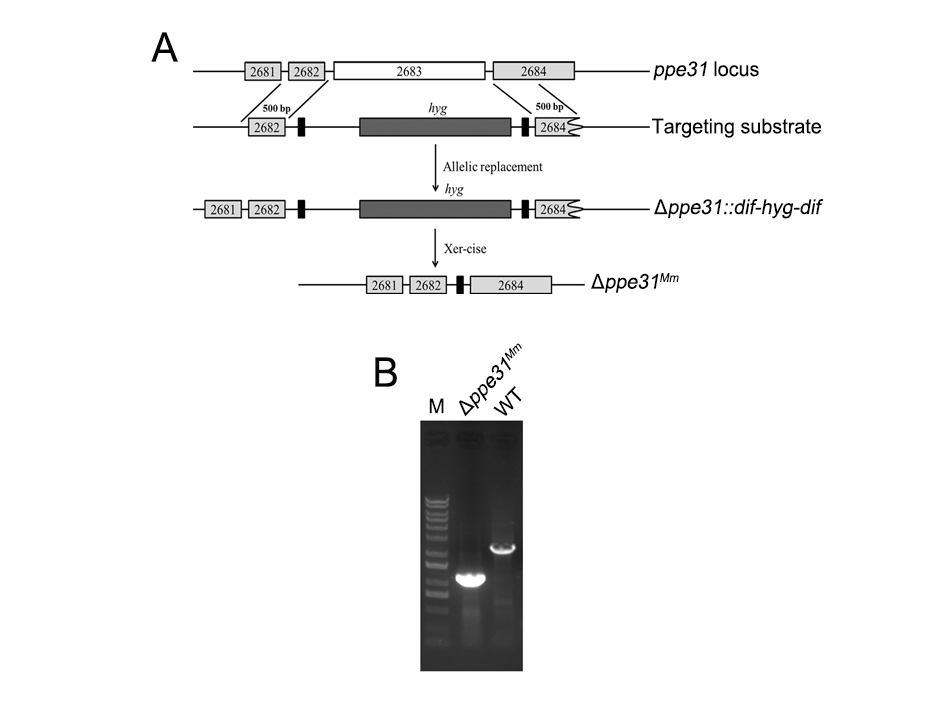

Supplement: Supplementary Figure 1 — Construction of the ppe31Mm mutant. Schematic representation of the strategy used for the generation of ppe31Mm gene replacement mutant (A). Confirmation of ppe31 Mm disruption in mutant. PCR amplifications were performed using specific primers. The result shows the PCR amplification gives a product of 2.2kb for wildtype M. marinum and 1kb for Δppe31Mm (B). [file Image_1.tif]

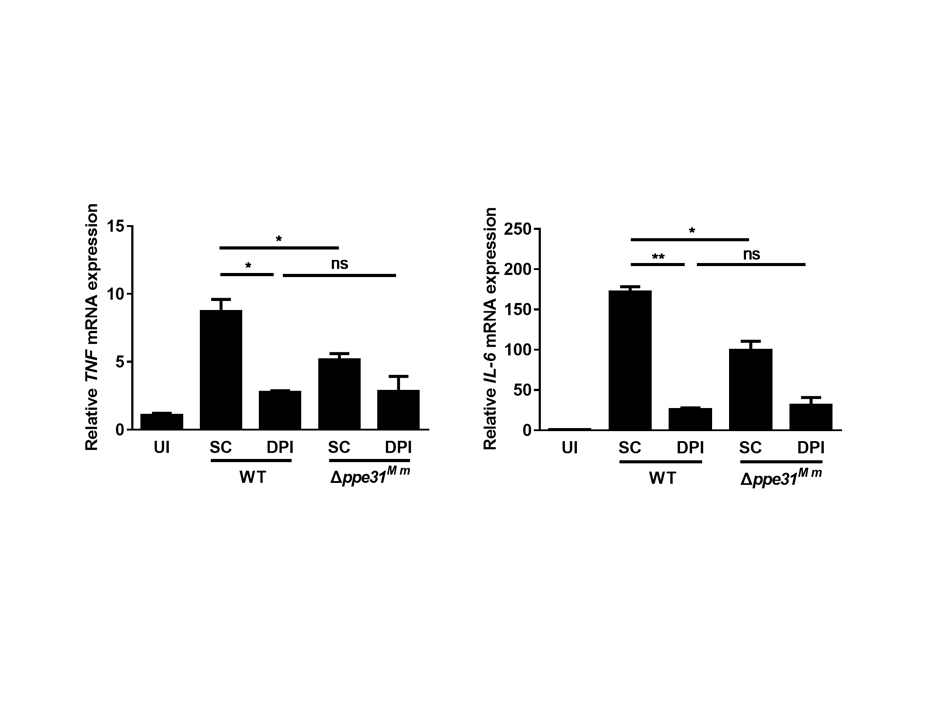

Supplement: Supplementary Figure 2 — PPE31 significantly increased the expression of inflammatory cytokines via reactive oxygen species in BMDMs. BMDMs were infected with WT, Δppe31Mm or comp-Δppe31Mm in the presence or absence in DPI (10μM). After 6h, the level of inflammatory cytokines was assessed by RT-qPCR for expressions of TNF-α and IL-6. WT, wild-type M. marinum, *p < 0.05, **p < 0.01. Data are shown as mean ± S.E.M. of three independent experiments. *p < 0.05, **p < 0.01. [file Image_2.jpeg]

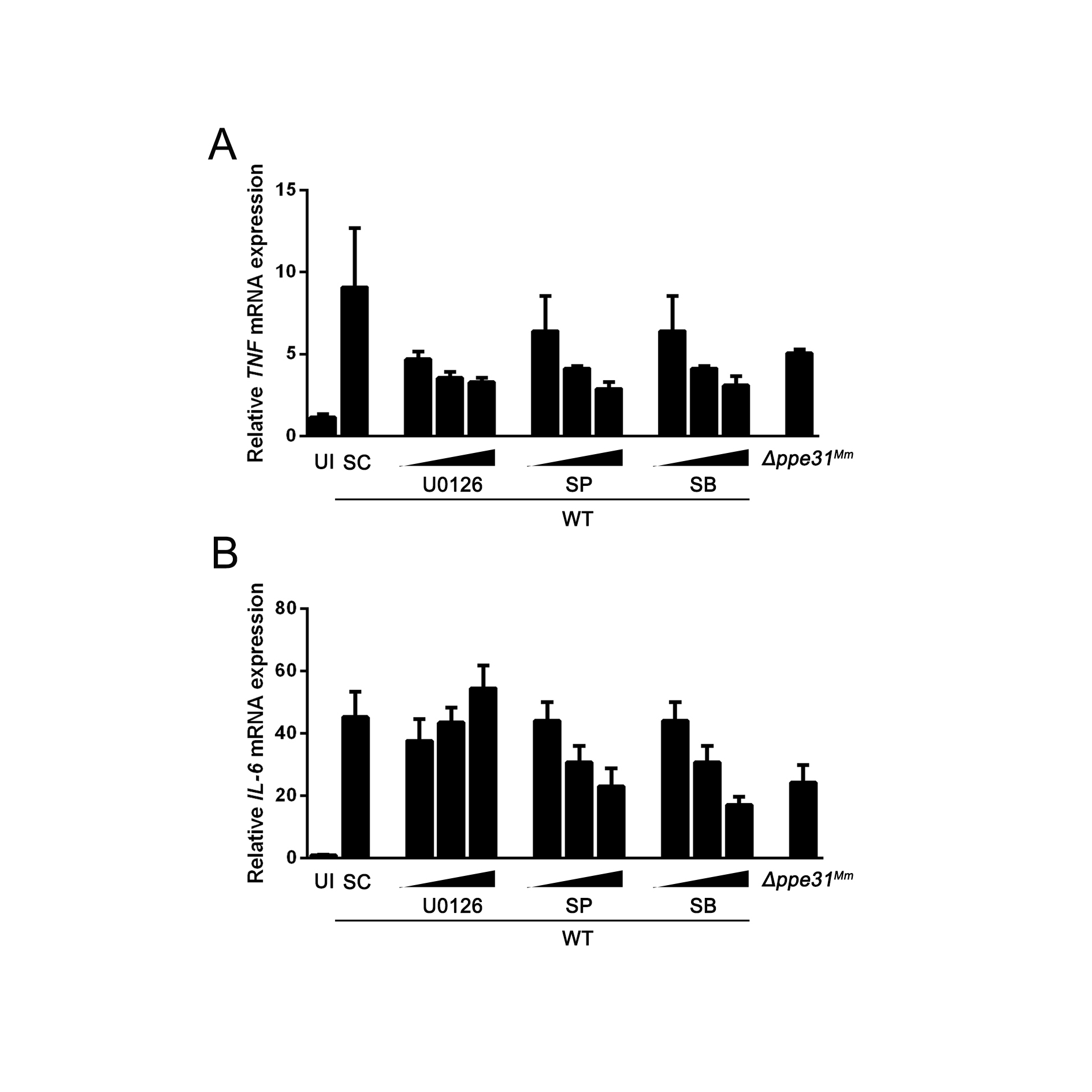

Supplement: Supplementary Figure 3 — PPE31 significantly increased the expression of inflammatory cytokines through JNK-dependent signaling. BMDMs were pretreated with U0126 (5, 10, 20 mM), SB203580 (SB; 1, 5, 10 mM), or SP600125 (SP; 5, 10, 20 mM) for 1h, and then infected with Δppe31Mm for1h, washed three times by PBS. After 6h, the level of inflammatory cytokines was assessed by real time PCR for expressions of TNF-α and IL-6. [file Image_3.jpeg]

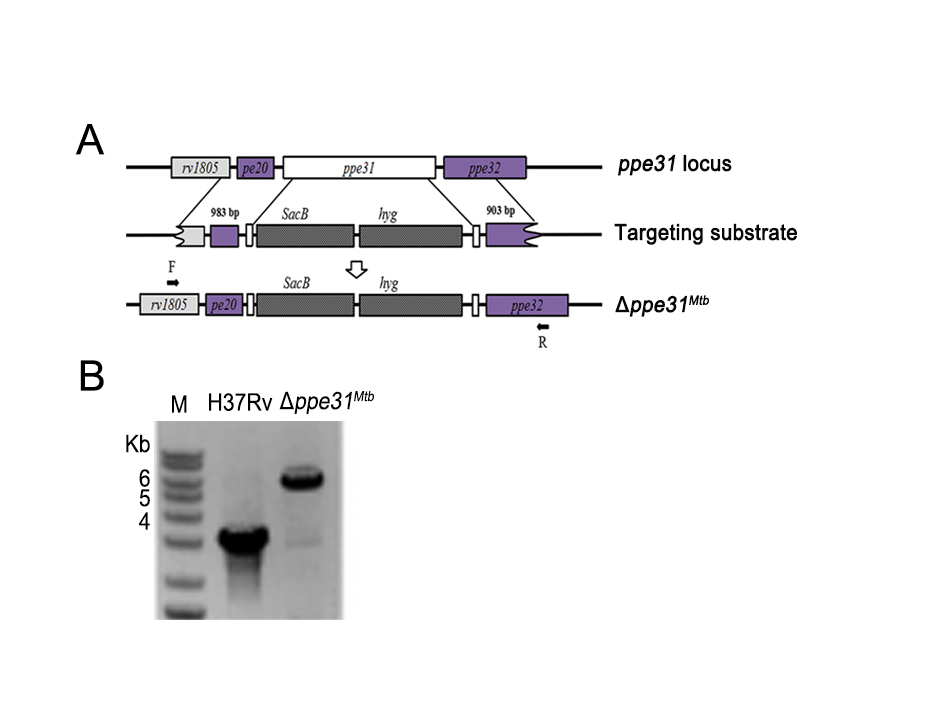

Supplement: Supplementary Figure 4 — Generation of ppe31Mtb gene replacement mutant. (A) Generation of M. tuberculosis ppe31Mtb gene replacement mutant. Schematic representation of the strategy used for the generation of ppe31Mtb gene replacement mutant. (B) Confirmation of ppe31Mtb disruption in mutant. PCR amplifications were performed using specific primers, using genomic DNA of H37Rv and Δppe31Mtb mutant. The result shows the PCR amplification with Rv ppe31-LL and Rv ppe31-RR pair, which gives a product of 3.2kb for H37Rv and 5.6kb for Δppe31. [file Image_4.jpeg]

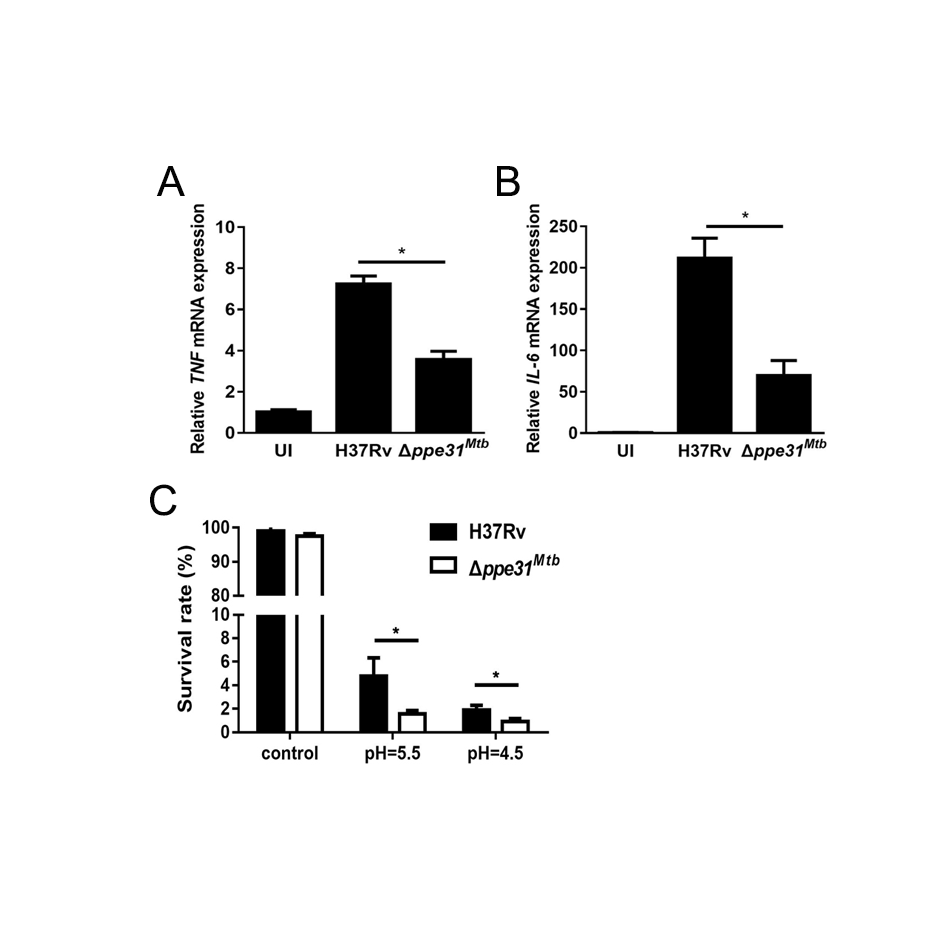

Supplement: Supplementary Figure 5 — RAW264.7 cells were infected with H37Rv or Δppe31Mtb (MOIs=10) for 6 h. The level expressions of inflammatory cytokines, TNF (A) and IL-6 (B) were assessed by RT-qPCR. The sensitivity of M. tuberculosis strains to acid condition. The bacteria grown to mid-log phase were collected by centrifugation and resuspended to the OD600 of 0.5 in 5 ml 7H9 (pH = 4.5 or 5.5). The results are percent survival post treatment with 7H9 medium (pH =4.5 or 5.5) for 7d (C). Data are shown as mean ± S.E.M. of three independent experiments. *p<0.05. UI, uninfected; M, marker. [file Image_5.tif]
